# Supplementary material for: Substrate recognition mechanism of the endoplasmic reticulum-associated ubiquitin ligase Doa10
Source: bioRxiv. 2024 Jan 9:2024.01.09.574907. Preprint. [Version 1] doi: 10.1101/2024.01.09.574907 (PMC10802466; doi:10.1101/2024.01.09.574907)
Supplement: Supplement 1 [file media-1.pdf]

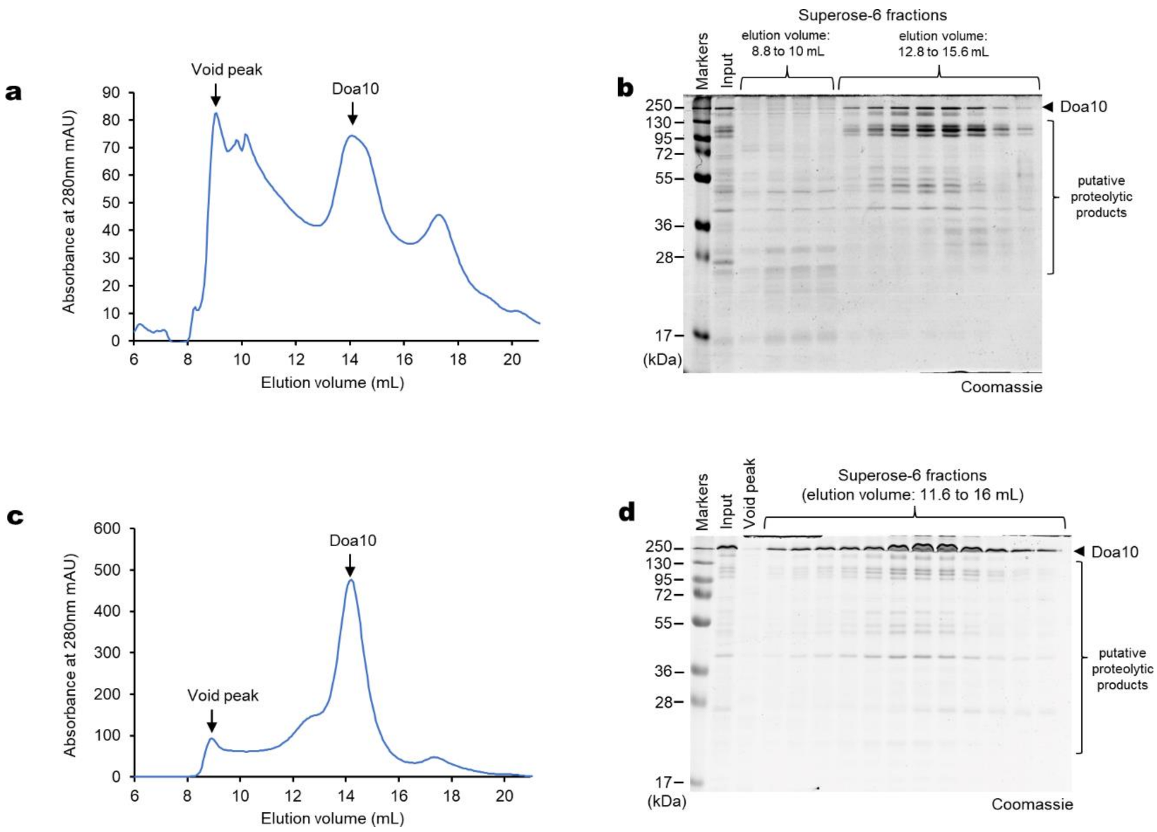

Supplementary Figure 1. Purification of Doa10 from *S. cerevisiae*.
a, Superose-6-increase size-exclusion chromatography (SEC) elution profile of affinity-purified endogenous Doa10 (GFP-tagged Doa10). **b**, Coomassie-stained SDS gel of Superose 6 fractions shown in **a**. **c** and **d**, As in **a** and **b**, but Doa10 was overexpressed by replacing the endogenous promoter with a *GAL1* promoter.

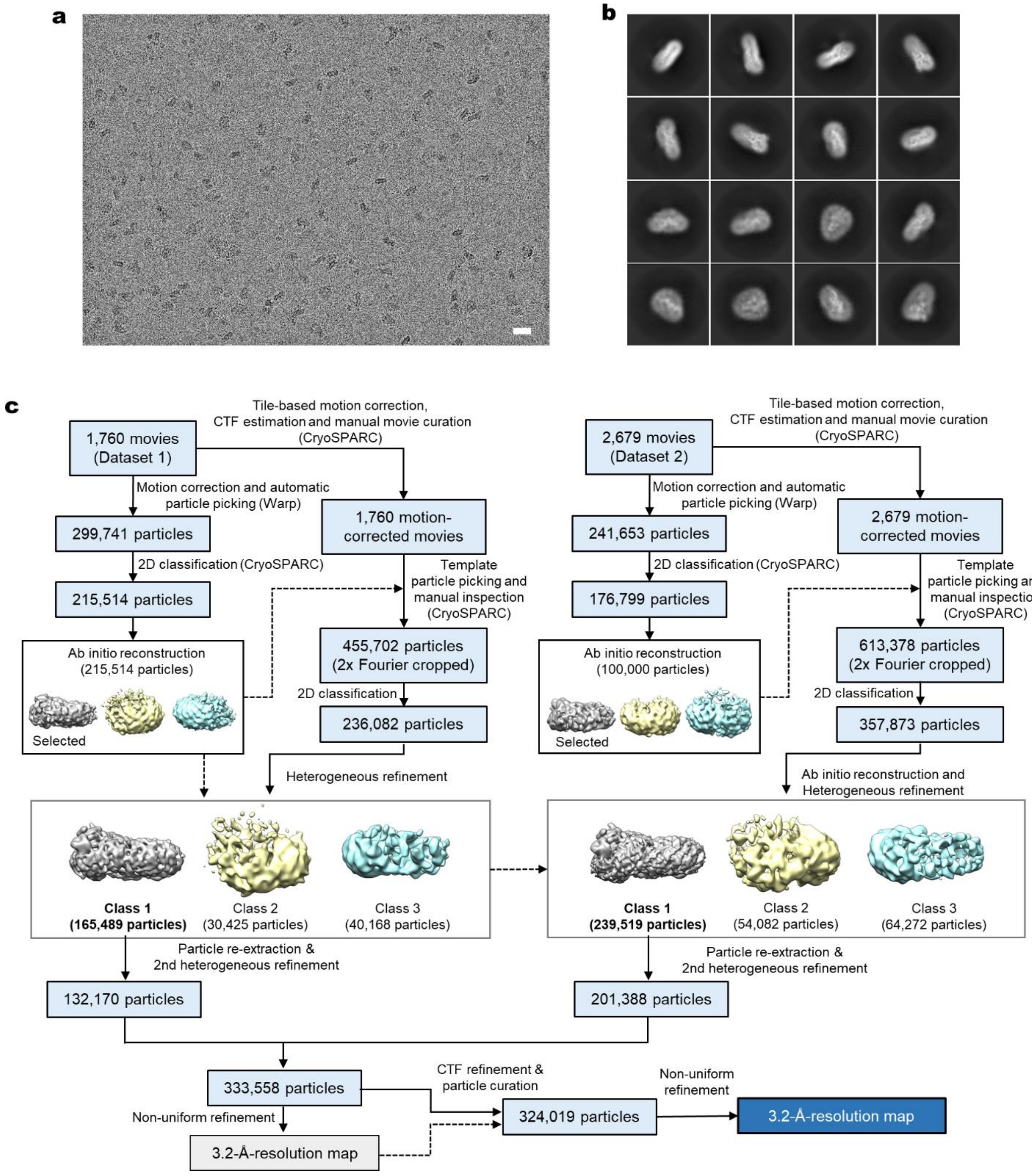

Supplementary Figure 2. Cryo-EM analysis of Doa10.

a, A representative micrograph (cropped) image of Doa10 particles. Scale bar, 200 Å. **b**, Representative 2D class averages. **c**, Schematic diagram for the cryo-EM image analysis procedure.

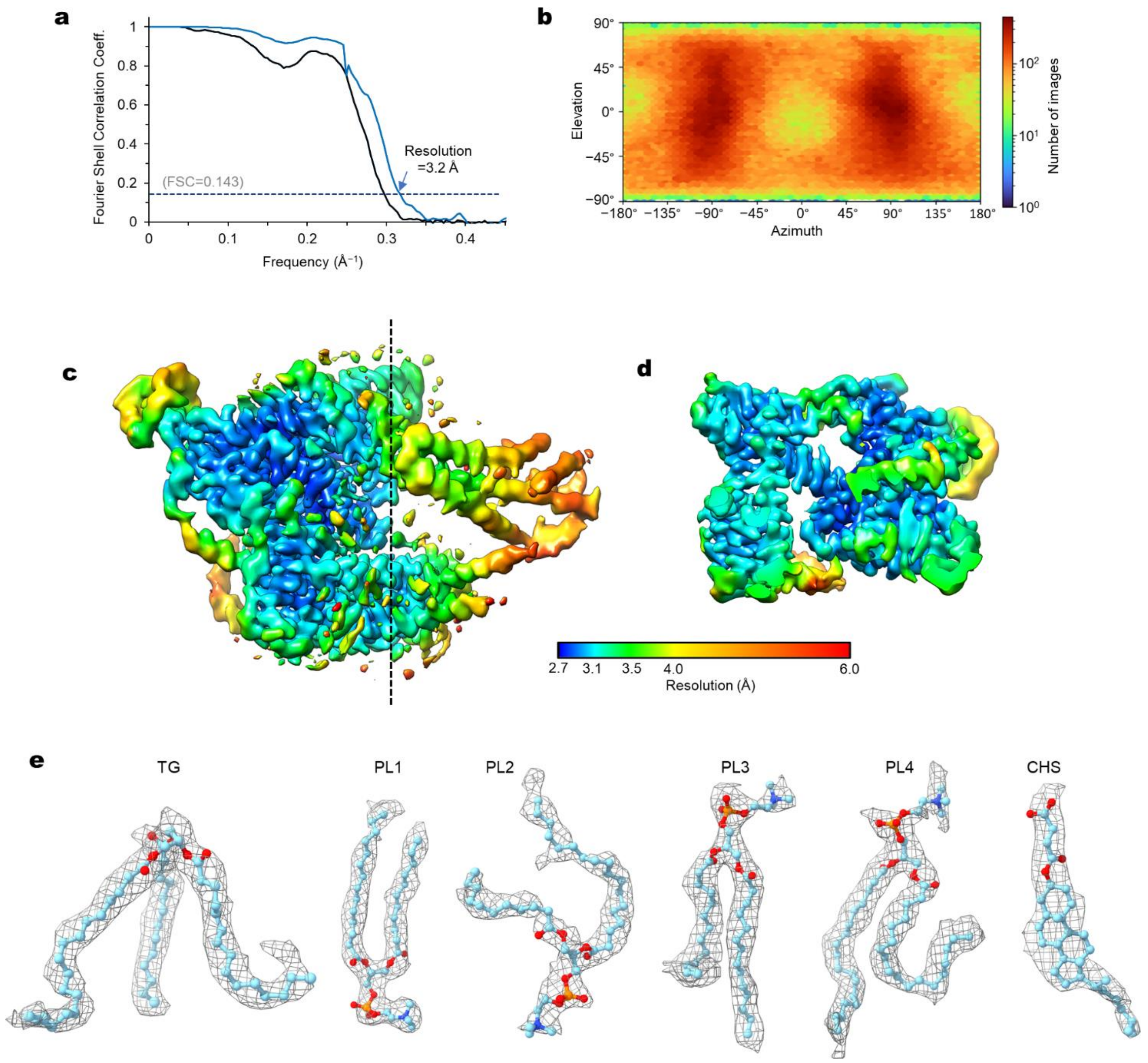

Supplementary Figure 3. Quality of the cryo-EM map of Doa10.
a, Fourier shell correlation (FSC) between the two half maps of the final 3D reconstruction. Blue, tight mask and corrected for masking; black, spherical mask. **b**, Distribution of particle orientation. **c** and **d**, Local resolution map (isosurface is an unsharpened map). The dash line indicates the cutaway plane for the view shown in **d**. **e**, Densities (gray mesh) of ordered lipids identified in the Doa10 cryo-EM structure.

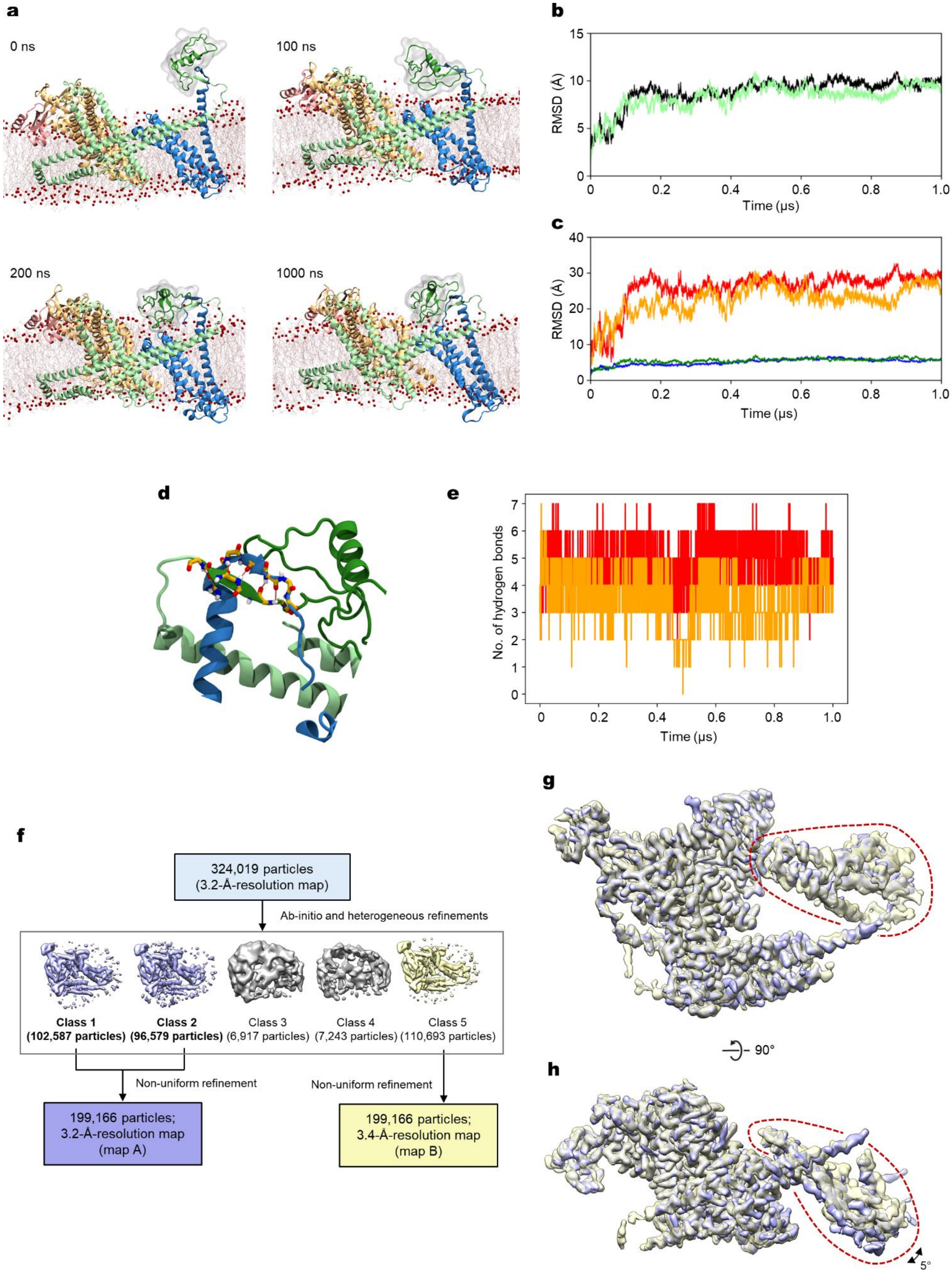

Supplementary Figure 4. Conformational flexibility of the RING-CH domain and CTD of Doa10.
(see next page for legend)

Supplementary Figure 4. Conformational flexibility of the RING-CH domain and CTD of Doa10.
a, Snapshots of MD simulations of wild-type Doa10 in a model membrane. The RING-CH domain is shown as a green ribbon and gray semi-transparent surface. **b**, Root-mean-square deviation (RMSD) using the Cα atoms for the two replicas of the Doa10 MD simulations. **c**, As in **b**, but additionally showing RMSD calculated for the RING-CH domain (red and orange) or the transmembrane domain (blue and green) for each replica. **d**, Snapshot of the MD simulations highlighting the two-strand β-sheet formed between the RING-CH and CTE of Doa10. **e**, The number of hydrogen bonds between the RING-CH and CTE over the duration of the MD simulations. Red and orange traces represent the two independent replicas. **f**, Additional classification of the Doa10 cryo-EM dataset to analyze the conformational heterogeneity of CTD. **g** and **h**, The two maps obtained from the procedure in **f** are overlaid. Shown are top (cytosolic; panel **g**) and side (panel **h**) views. CTD is indicated by red dashed lines.

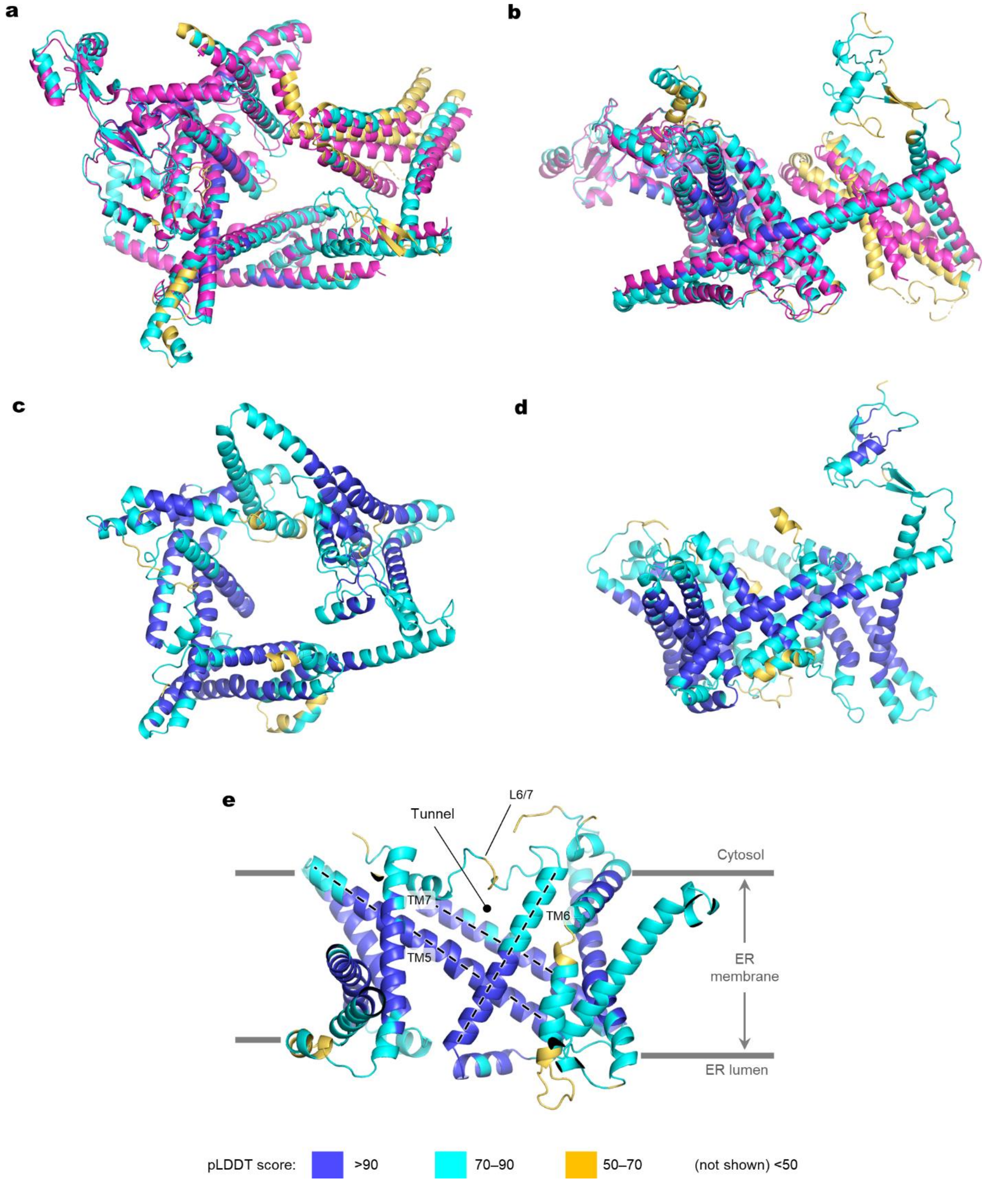

Supplementary Figure 5. AlphaFold2 models of yeast Doa10 and human MARCH6.
a and **b**, Comparison between the cryo-EM structure (magenta) and AlphaFold2 model (other colors) of yeast Doa10. The AlphaFold2 model is colored according to the pLDDT score (blue, >90; cyan, 70 to 90; yellow, 50 to 70). Parts below less than a pLDDT score below 50 were not shown. Shown are top (cytosolic; panel **a**) and side (panel **b**) views. **c**–**e**, AlphaFold2 model of human MARCH6. Top (**c**) and side (**d**) views, and a view into the lateral tunnel from the central cavity (**e**).

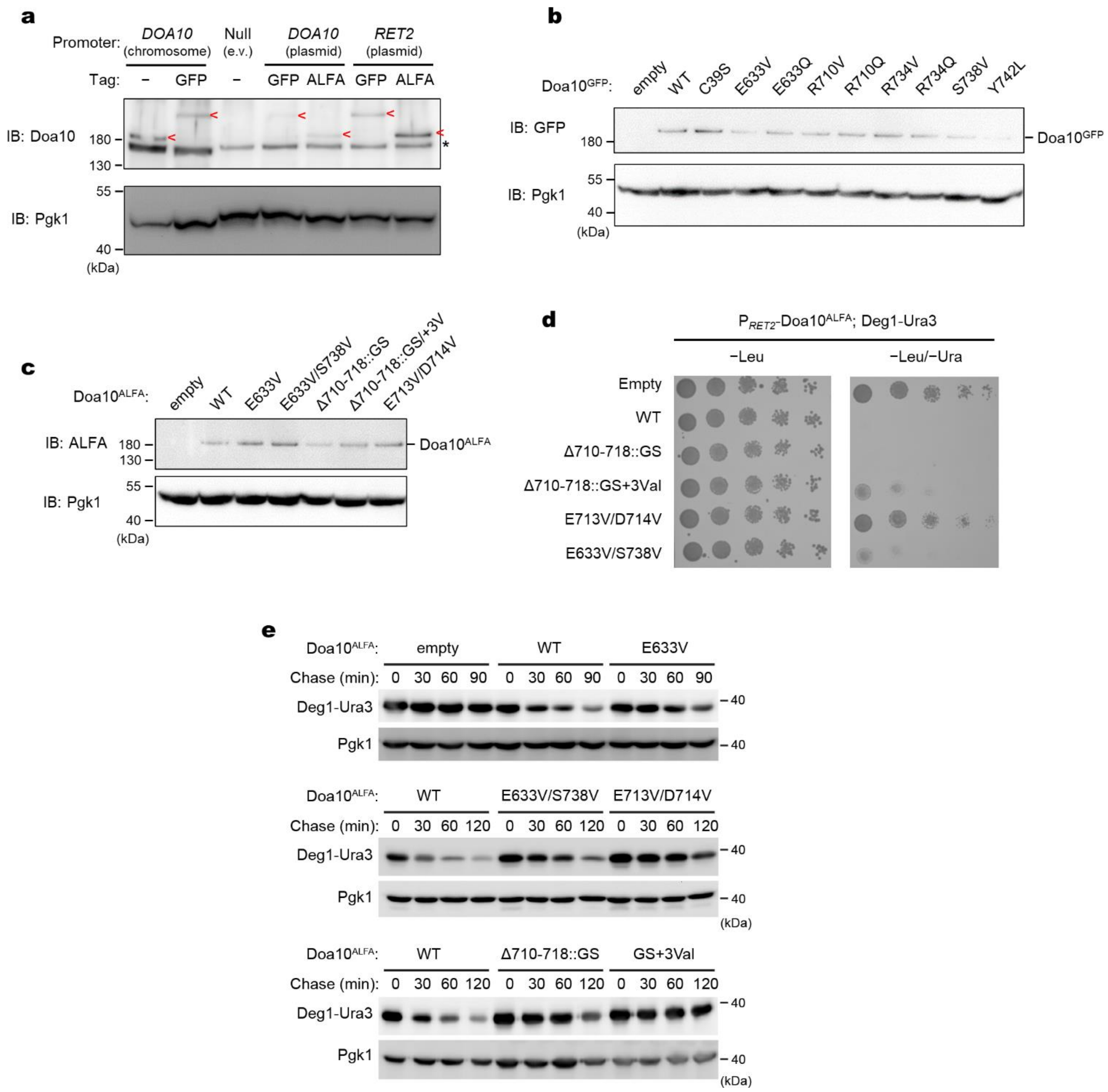

Supplementary Figure 6. Effects of Doa10 mutations on Deg1-Ura3 degradation.
a, Comparison of the endogenous and exogenous expression levels of Doa10 with either a C-terminal GFP-tag or ALFA-tag. Protein levels were measured by anti-Doa10 immunoblotting. 3-phosphoglycerate kinase (Pgk1) was used for a loading control. **b** and **c**, Expression levels of WT Doa10 and indicated mutants with a C-terminal GFP-tag (panel b) or with a C-terminal ALFA tag (panel c) was measured by immunoblotting. All the variants were expressed from a Doa10 promoter in a CEN/ARS plasmid. Pgk1 was used for a loading control. **d**, Yeast growth inhibition assay was performed as in Fig. 4e, but expressing Doa10 under the *RET2* promoter. Note that a higher expression level of Doa10 under the *RET2* promoter compared to the one under the endogenous Doa10 promoter (panel a) produces an overall stronger growth inhibition. **e**, Cycloheximide chase analysis of Deg1-Ura3 with indicated Doa10 mutants. Deg1-Ura3 levels were detected by anti-Strep-tag immunoblotting. Pgk1 was used for a loading control. See Fig. 4f for mean±s.e.m. from three independent experiments.

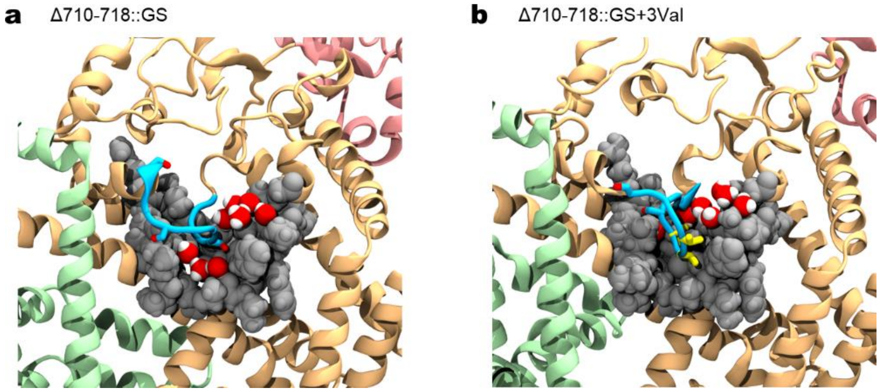

Supplementary Figure 7. Example snapshots of MD simulations on Doa10 L6/7 mutants.
As in Fig. 4 g–i, but showing the Δ710-718::GS and Δ710-718::GS+3Val mutants of Doa10.

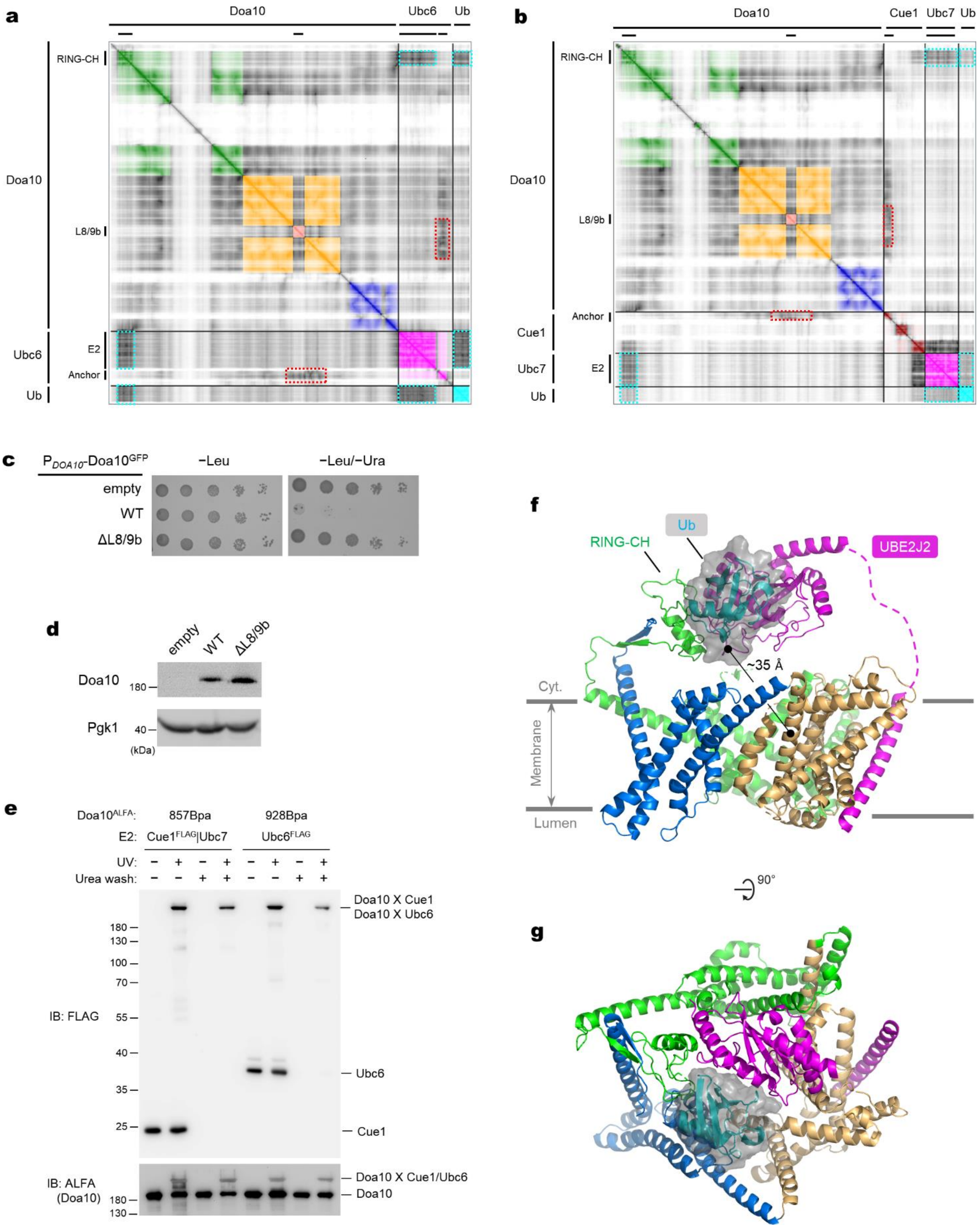

Supplementary Figure 8. Interactions between Doa10 and E2s.
(see next page for legend)

Supplementary Figure 8. Interactions between Doa10 and E2s.

a, Predicted Aligned Error (PAE) matrix for the AlphaFold2 model of the Doa10–Ubc6–Ub complex. Regions are colored according to the domain color scheme in Fig. 6 (only regions with >50 pLDDT score are in color). Dashed boxes in cyan indicate the regions corresponding to contacts between the RING-CH, E2 domain, and Ub. Dashed boxes in red indicate the contacts between the TM anchor of Ubc6 and Doa10. **b**, As in **a**, but for AlphaFold2 model for Doa10–Cue1–Ubc7–Ub. **c**, Yeast growth inhibition assay comparing the activities of WT and ΔL8/9b Doa10. Doa10 with a C-terminal GFP-tag were expressed from a *DOA10* promoter in a CEN/ARS plasmid. **d**, Expression levels of WT and ΔL8/9b Doa10 were measured by anti-GFP immunoblotting. Pgk1 was used as a loading control. **e**, Verification of covalent, UV-dependent crosslink adducts. Where indicated, ALFA nanobody beads were additionally washed with 6 M urea during immunoprecipitation to dissociate non-covalently associated proteins. **f** and **g**, As in Fig. 6 a and b, but showing an AlphaFold2 model of a complex of human MARCH6, UBE2J2, and Ub. Panels in **f** and **g** show side and cytosolic views, respectively.

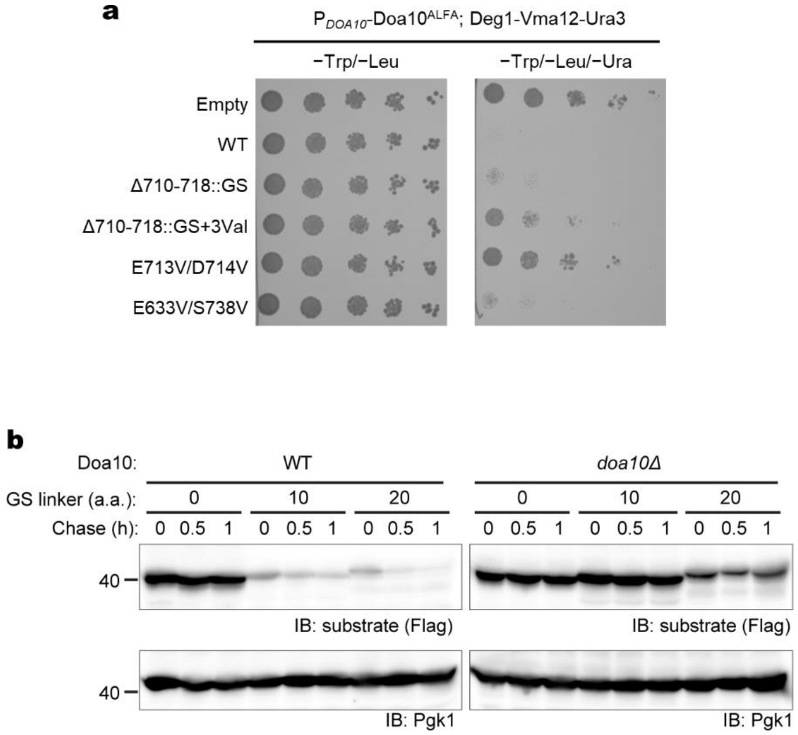

Supplementary Figure 9. Doa10-dependent degradation of Deg1-Vma12-Ura3.
a, Yeast growth inhibition assay with Deg1-Vma12-Ura3 and indicated Doa10 mutants. Deg1-Vma12-Ura3 was expressed under the *MET25* promoter, and all Doa10 mutants were expressed from the *DOA10* promoter. **b**, Cycloheximide chase analysis of $Deg1_{1\text{-}35}$-$Vma12_{\Delta 132}$-Ura3 with different lengths of the GS linker between $Deg1_{1\text{-}35}$ and $Vma12_{\Delta 132}$. Deg1 substrates were detected by anti-FLAG-tag immunoblotting (a FLAG-tag is attached to the C-terminus of Ura3). Pgk1 was used for loading control. See Fig. 7d for mean±s.e.m. from three independent experiments.

Table S1. Cryo-EM data collection, refinement and validation statistics.

	**Doa10 from *S. cerevisiae*** (PDB:8TQM, EMDB: 41508)
**Data collection and processing**	
Microscope	FEI Titan Krios G2
Magnification	64,000x
Voltage (kV)	300
Electron exposure (e⁻/Å²)	50
Defocus range (μm)	−0.7 to −2.4
Pixel size (Å)	0.91
Symmetry imposed	*C*1
Initial particle images (no.)	541,394
Final particle images (no.)	324,019
Map resolution (Å)	3.2
FSC threshold	0.143
Map resolution range (Å)	2.72 (highest), 7.15 (75% percentile)
**Refinement**	
Initial model used	De novo + AlphaFold2 model
Model resolution (Å)	3.3
FSC threshold	0.5
Map sharpening *B* factor (Å²)	112
Model composition	
Non-hydrogen atoms	7,231
Protein residues	843
Ligands	7
B factors (Å²)	
Protein	34.10
Ligand	12.05
R.m.s. deviations	
Bond lengths (Å)	0.005
Bond angles (°)	1.055
**Validation**	
MolProbity score	1.70
Clashscore	12.0
Poor rotamers (%)	0
Ramachandran plot	
Favored (%)	97.47
Allowed (%)	2.53
Disallowed (%)	0
CaBLAM outliers (%)	0.73

Table S2. List of yeast strains

Name	Genotype	Reference
MHY4086	*MATa his3-Δ200 ura3-52 trp1-Δ63 leu2-3112 lys2-801::LYS2::*Deg1-Ura3 *doa10Δ::HphMX4*	Stuerner et al., 2012
JY103	*MAT***a** *his3-Δ200 ura3-52 trp1-Δ63 leu2-Δ1 lys2-801 ADE2*	Laney et al., 2006
MHY10818	*MATα his3-Δ200 leu2-Δ1 ura3-52 lys2-801 trp1-Δ63 doa10Δ::hphMX4*	Mehrtash et al., 2022
BY4741	*MATa his3-1, leu2-0, met15-0, ura3-0*	Horizon Discovery
ySI-118	BY4741 *DOA10*-TEV-GFP::*natMX*	This study
ySI-154	BY4741 *leu2*::P_{GAL1}-Doa10-TEV-GFP::*natMX*	This study
ySI-167	BY4741 *doa10Δ*::*natMX*	This study
ySI-266	MHY10818 *cue1Δ*::*natMX*	This study
yKW-283	ySI-167 *leu2*::P_{PGK1}-Deg1-Ura3-2xstrep::*hphMX4*	This study
yYC-307	MHY10818 *ubc6Δ*::*natMX*	This study

Table S3. List of plasmids

Name	Description	Reference
pYTK001 to 096	Original MoClo YTK parts	Lee et al., 2015
pYTK-e102	*LEU2* integration vector containing a hygromycin marker (assembled from pYTK008, pYTK047, pYTK073, pYTK079, pYTK087, pYTK090, and pYTK093)	This study
pYTK-e205	MoClo YTK part (type 4a) for 2x-Strep (Amino acid sequence: SGWSHPQFEKGGGSGGGSGGSAWSHPQFEK*)	This study
pYTK-e111	CEN/ARS plasmid containing a Ura3 marker (assembled from pYTK008, pYTK047, pYTK073, pYTK074, pYTK081, and pYTK084)	This study
pYTK-e112	CEN/ARS plasmid containing a Leu2 marker (assembled from pYTK008, pYTK047, pYTK073, pYTK075, pYTK081, and pYTK084)	This study
pDoa10-Doa10-split1	Split pYTK-e112 (CEN/ARS)-P_{DOA10}-Doa10(1-608)	This study
pRET2-Doa10-split1	Split pYTK-e112 (CEN/ARS)-P_{RET2}-Doa10(1-608)	This study
pTDH3-Doa10-split1	Split pYTK-e112 (CEN/ARS)-P_{TDH3}-Doa10(1-608)	This study
Doa10–GFP-split2	Split pYTK-e112 (Leu2 marker)-Doa10(608-1319)-TEV-GFP (Amino acid sequence of the tag: GTGSGTGENLYFQGTASGGGSKGEELFTGVVPILVELDGDVNG HKFSVSGEGEGDATYGKLTLKFICTTGKLPVPWPTLVTTFGYGV QCFARYPDHMKQHDFFKSAMPEGYVQERTIFFKDDGNYKTRAE VKFEGDTLVNRIELKGIDFKEDGNILGHKLEYNYNSHNVYIMADK QKNGIKVNFKIRHNIEDGSVQLADHYQQNTPIGDGPVLLPDNHYL STQSALSKDPNEKRDHMVLLEFVTAAGITHGMDELYKVDLDK*)	This study
Doa10-ALFA-split2	Split pYTK-e112 (Leu2 marker)-Doa10(608-1319)-ALFA (Amino acid sequence of the tag: GTSRLEEELRRRLTE*)	This study
pKW043	MoClo YTK part (type 3a) for Deg1	This study
pKW050	MoClo YTK part (type 3b) for Ura3	This study
pKW155	pYTK-e102-PGK1-Deg1-Ura3-2xstrep. Assembled from pYTK-e102, pYTK011 (P_{PGK1}), pKW043 (pYTK001-Deg1), pKW050 (pYTK001-Ura3), pYTK001-e205 (2xStrep), pYTK061 (tENO1)	This study
p414-Deg1-Vma12-Ura3	Plasmid expressing Deg1-Vma12-Ura3 from a *MET25* promoter	Ravid et al. 2006
pSK-B399-GFP-NAT	Template plasmid for PCR to generate a DNA fragment containing a C-terminal TEV-GFP tag and natMX (for chromosomal tagging).	Gift from the S. Klinge lab
pYC-300	pYTK-e111-P_{GAL1}-Cue1-2xFLAG :: P_{GAL1}-Ubc7-2xStrep	This study
pYC-301	pYTK-e111-P_{GAL1}-3xFLAG-6xHis-Ubc6	This study
pYC-302	pYTK-e111-P_{GAL1}-Cue1-2xFLAG :: P_{GAL1}-Ubc7-2xSPOT :: P_{GAL1}-3xFLAG-6xHis-Ubc6	This study

Supplementary Movie 1. MD simulation on the cryo-EM/AlphaFold2 hybrid model of Doa10.
The hybrid model was placed in a model lipid bilayer, and an all-atom MD simulation was performed for a 1-µs duration. Shown is the side view along the membrane plane with Doa10 in a ribbon representation and positions of lipid headgroups indicated by red spheres. The color scheme is the same as in Fig. 1. The hybrid model includes the N-terminal RING-CH domain, for which an additional surface representation (surface in transparent gray) is used.

Supplementary Movie 2. Zoom-in views into the lateral tunnel of WT and mutant Doa10 in MD simulations.
Parts of TMs 5 to 7 that form the lateral tunnel are shown (front view). In the WT, E633V/S73V, and E713V/D714V panels, the L6/7 loop (positions 710-718) is shown in cyan licorice, whereas in the Δ710-718::GS (‘GS’) and Δ710-718::GS+3Val (‘GS+3Val’) mutants, the equivalent segment is shown in blue and green licorice, respectively. Gray spheres are used for a space-filling representation of the wedge-shaped tunnel-lining amino acids from TMs 5 to 7. Yellow residues are mutated valine.
